# Brillouin and Raman Micro-Spectroscopy: A Tool for Micro-Mechanical and Structural Characterization of Cortical and Trabecular Bone Tissues

**DOI:** 10.3390/ma14226869

**Published:** 2021-11-14

**Authors:** Martina Alunni Cardinali, Assunta Morresi, Daniele Fioretto, Leonardo Vivarelli, Dante Dallari, Marco Govoni

**Affiliations:** 1 Department of Physics and Geology, University of Perugia, Via A. Pascoli, I-06123 Perugia, Italy; daniele.fioretto@unipg.it; 2 Department of Chemistry, Biology and Biotechnology, University of Perugia, Via Elce di Sotto 8, I-06123 Perugia, Italy; assunta.morresi@unipg.it; 3 CEMIN—Center of Excellence for Innovative Nanostructured Material, I-06123 Perugia, Italy; 4 Reconstructive Orthopaedic Surgery and Innovative Techniques—Musculoskeletal Tissue Bank, IRCCS Istituto Ortopedico Rizzoli, Via G.C. Pupilli 1, 40136 Bologna, Italy; leonardo.vivarelli@ior.it (L.V.); dante.dallari@ior.it (D.D.); marco.govoni@ior.it (M.G.)

**Keywords:** Raman spectroscopy, Brillouin spectroscopy, bone material characterization, bone micro-scale architecture

## Abstract

Human bone is a specialized tissue with unique material properties, providing mechanical support and resistance to the skeleton and simultaneously assuring capability of adaptation and remodelling. Knowing the properties of such a structure down to the micro-scale is of utmost importance, not only for the design of effective biomimetic materials but also to be able to detect pathological alterations in material properties, such as micro-fractures or abnormal tissue remodelling. The Brillouin and Raman micro-spectroscopic (BRmS) approach has the potential to become a first-choice technique, as it is capable of simultaneously investigating samples’ mechanical and structural properties in a non-destructive and label-free way. Here, we perform a mapping of cortical and trabecular bone sections of a femoral epiphysis, demonstrating the capability of the technique for discovering the morpho-mechanics of cells, the extracellular matrix, and marrow constituents. Moreover, the interpretation of Brillouin and Raman spectra merged with an approach of data mining is used to compare the mechanical alterations in specimens excised from distinct anatomical areas and subjected to different sample processing. The results disclose in both cases specific alterations in the morphology and/or in the tissue chemical make-up, which strongly affects bone mechanical properties, providing a method potentially extendable to other important biomedical issues.

## 1. Introduction

Human lamellar bone is a complex material, designed in the form of a hierarchical structure with several levels of organization going from the macroscale down to the nanoscale [1,2,3]. At the macroscopic level, the structural framework of the bone tissue can be classified into two types, namely cortical and trabecular bone, which differ in their anatomic distribution, function, and lamellar organization. The cortical tissue, constituting the envelope of the whole-bone structure, is formed by the so-called Haversian systems or osteons, cylindrical structures in which the lamellae are concentrically organized around central blood vessels. On the other hand, the trabecular tissue in the inner portion of bones is composed of adjacent lamellae forming bony spicules—i.e., trabeculae—that encapsulate the bone marrow [4]. At the microscopic level, the lamellar structure of both osteons and lamellae are constituted by an extracellular mineralized matrix (ECM) organized in the form of both an ordered phase of mineralized collagen bundles, responsible for lamellar strength and resistance, and a disordered phase, filling the spaces among the fibres and allowing for the survival of cells [5].

Finally, at the sub-micro and nanoscale, the mineralized collagen bundles are composite structures formed in turn by smaller fibrils of collagen type I, reinforced by carbonated hydroxyapatite nanocrystals.

A hierarchical organization is often the preferred choice by nature to built-up materials with complex collective properties from simpler constituents—i.e., biological macromolecules [6]. However, an important consequence is that an impairment in the correct distribution of these components at the lower scales can have important effects on the chemical and mechanical properties of the structure as a whole. In that sense, deeply knowing the structural properties of the material bone already at the submicrometric level is an important issue to address in biomedical sciences, since the modelling and the design of effective new biomaterial cannot disregard the ability to mimic the characteristics of the native human tissue [7,8]. Therefore, developing material as similar as possible to the host bone is recommended for allowing the best biological behaviour [9].

Brillouin and Raman micro-spectroscopy (BRmS) is an optical correlative method for material characterization at the microscale based on light scattering [10,11]. Specifically, Brillouin spectroscopy describes the mechanical properties by means of the interaction of light with acoustic waves, spontaneously propagating in the matter due to thermal motions [12,13,14]. On the other hand, Raman spectroscopy is a well-established vibrational technique for the chemical characterization of organic material [15]. In our spectroscopic setup, they are paired together with a confocal microscope for imaging and mapping purposes with micrometric resolution [16]. Brillouin and Raman micro-spectroscopy has provided valuable results in the structured biomaterial description, as well as in tissue characterization in both healthy and pathological conditions [17,18,19,20,21,22]. Therefore, it has the great advantage of establishing correlations between alterations in the sample’s mechanical properties and chemical composition. Moreover, it is contactless, not destructive, and label-free, thus having the potential for in vivo application via endoscopic probes [23,24].

In our previous studies, BRmS turned out to be a precious tool for musculoskeletal tissue analysis, revealing the existence of a complex micro-architecture, in both the articular cartilage of a femoral epiphysis and bone tissue structures of a femoral diaphysis. Specifically, Brillouin imaging was able to detect the coexistence of two distinct phases in the extracellular matrix organization, retaining the dimension of aggregates of sub-metric size. The addition of a correlated Raman analysis allows us to assign these two components to the presence in the same scattering volume of an ordered phase of collagen bundles and a not-ordered phase of poorly oriented and mineralized collagen fibrils, non-collagenous proteins, and water. Whereas the first one is responsible for bone and cartilage tissue resistance to the tensile and compressive stresses, the latter is fundamental to producing a more accessible environment for metabolite diffusion and cellular growth and survival [25,26].

Here, we perform BRmS imaging of both healthy cortical and trabecular bone tissue types, employing a section excised from the infero-medial region of the femoral epiphysis. It is worth noting that the characterization of different healthy tissue types coming from distinct anatomical regions provides the basis for any further application of the technique to the detection of chemo-mechanical modifications of bone micro-architecture, due to sample processing or to the onset of pathological conditions, as well as to the characterization of bio-inspired material and prosthetic integration inside the native tissue. Concerning this, we present also the first application of the technique to compare the chemo-mechanics of bone tissue when subjected to different types of sample processing, i.e., freezing at −80 °C and fixation in paraformaldehyde (PFA) followed by ethanol storage, and when excised from distinct anatomical portions of the human femur, i.e., femoral epiphysis and diaphysis. Traditional spectroscopic data analysis approaches were supported by the employment of a multivariate statistical approach—i.e., principal component analysis (PCA)—revealing the capability of data mining techniques to successfully recognize patterns and trends in the spectroscopic dataset useful for classification purposes [27,28,29].

## 2. Materials and Methods

### 2.1. Samples Description and Preparation

Brillouin and Raman analyses were performed on two femoral cross-section diaphyses and one femoral head portion procured from three different cadaver donors by the accredited public non-profit Musculoskeletal Tissue Bank of IRCCS Istituto Ortopedico Rizzoli (Bologna, Italy; EU TE Code: IT000096). In this study, only specimens not suitable for transplantation and considered as waste materials were used, according to the Italian Legislation and National Transplant Centre’s guidelines. After the sample processing procedures performed in a Good Manufacturing Practice (GMP)-Class A clean room environment, one cross-section diaphysis (a 5 mm thick ring with a 2.5 diameter collected from the lesser trochanter) and the femoral head portion (about 1 cm in thickness and 1 cm in width) were washed with sterile physiological saline solution and stored at −80 °C until experimental analysis. On the other hand, the second femoral cross-section (same dimension and anatomical region of the abovementioned cross-section diaphysis) was fixed in 4% buffered paraformaldehyde (PFA; Sigma-Aldrich, Milan, Italy) for 24 h, washed in running tap water and distilled water, and stored at room temperature in a 70% alcohol/water mixture. Before measurements, the sample was removed from ethanol and air-dried at room temperature.

**Figure 1:**
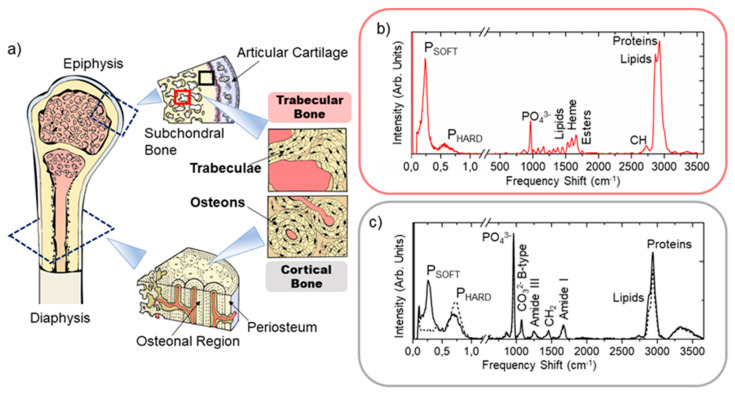
(**a**) Graphical sketch of the human femoral bone describing anatomical regions of interest (i.e., the epiphysis and the diaphysis, with their macroscopic organization in cortical bone and trabecular bone tissues). Black square: cortical bone; red square: trabecular bone. Typical Brillouin (before the break) and Raman (after the break) spectra were collected in (**b**) the trabecular bone (red) and in (**c**) the cortical bone (black dashed line—extracellular matrix portion; black solid line—blood vessels portion) of the frozen femoral epiphysis, reporting the signatures of characteristic peaks.

The main histological characteristics of cortical and trabecular bone tissues are shown in the graphical sketch of Figure 1a. Their organization differs macroscopically for the anatomical distribution and physiological functions and microscopically for the lamellar spatial disposition. Specifically, cortical bone constitutes the rigid capsule of the whole bone structure in the diaphysis and the epiphysis and is responsible for the skeleton sustenance and mechanical resistance to hurt. Trabecular bone is present inside the end of long bones—i.e., the epiphysis—and in the medullary cavity, where it acts as a support network for the containment and protection of our hematopoietic organ—i.e., the bone marrow. Moreover, it is also involved in the homeostasis of calcium and phosphate ions, and thus more involved in metabolic bone diseases, such as osteoporosis, osteomalacia, fluorosis, or primary hyperparathyroidism. From the microscopic point of view, the cortical bone (black square—Figure 1a) is organized in concentric lamella to form the so-called osteon or Haversian system, while in the trabecular bone (red square), the lamellae are settled adjacent one to the other to constitute bony spiculae—i.e., the trabeculae. The spaces among the trabeculae are filled by the bone marrow constituents [4].

### 2.2. Brillouin and Raman Micro-Spectroscopic Setup

The BRmS consists of a 532 nm single-mode solid-state laser, a polarizing beamsplitter, which reflects the laser light into a microscope objective lens for sample imaging. Then, the backscattered light from the sample is collected by the same objective and then split in frequency and direction by an edge filter, so that the Stokes component (>30 cm^−1^) is sent to a Horiba iHR320 Triax Raman monochromator (Kyoto, Japan), and the quasi-elastic and anti-Stokes components are sent to a high contrast multipass tandem Fabry–Perot interferometer (TFP-2 HC, JRS Scientific Instruments, Zürich, Switzerland). The sample is mounted on an xyz translation stage for mapping and imaging. More details of the instrumentation can be found in [16,26].

The microscope objective selected for the measurements of bone sections is a 20× objective (NA 0.42) so that a volume of about 2 μm in diameter for about 10 μm in-depth, corresponding to a portion of a single lamella, is enlightened by the laser beam. The measurements were acquired in regions of interest (ROIs) in both trabecular and cortical areas of each sample. Laser power was filtered to about 7 mW to avoid tissue photodamage, and the acquisition time was set to 1 s and 55 accumulations for each spectrum. Maps were acquired considering a 3 μm step in both x and y axes.

### 2.3. Brillouin Data Analysis

Brillouin light scattering deals with the interaction of the incident light with acoustic waves, spontaneously propagating in the matter thermally activated [10,13]. In the Brillouin spectra, the peaks’ intensity is directly dependent on the concentration of the elastic species supporting the acoustic mode, whereas the square of its Brillouin frequency shift, ν_B_, is related to the elastic species hardness, expressed by the longitudinal elastic modulus M:

(1) $$M=\nu_{B}^{2}{\;\lambda }^{2}\rho /4n^{2}$$

where ρ is the mass density, n the refractive index of the medium, and λ the wavelength of the laser source.

Typical Brillouin spectra collected in bone tissue are reported in Figure 1b,c (before the break), revealing the existence of at least two well-separated peaks for the same scattering volume, namely P_SOFT_ (between 4 and 13 GHz) and P_HARD_ (between 13 and 32 GHz), and thus confirming the coexistence of micrometric aggregates with a different mechanical identikit in all the three samples [30,31]. In our previous paper, peaks’ intensity and frequency shift values were calculated by computing the spectral moment of P_SOFT_ and P_HARD_ peaks of the Brillouin spectrum, as described in [25,26,32]. This approach has proven to be particularly well suited for obtaining average information on extremely heterogeneous regions of bone tissue requiring only fixing the range extremes. Specifically, we estimated the average frequency shift of each peak through the calculation

(2) $${\bar{\nu }}_{B}=\sum_{i}I_{i}\upsilon_{i}/\sum_{i}I_{i}$$

where I_i_ is the intensity, and the index i spans spectral channels in the range 4–13 GHz for the low-frequency (ν_SOFT_) and 13–32 GHz for the high-frequency (ν_HARD_) modes. Finally, since the intensity of the peaks is dependent upon the quantity of material enlightened by the laser beam in each point of the sample, and the bone tissue is characterized by a rough surface, a normalization procedure is required to estimate the relative ratio of the volume occupied by the two components from one point to the other. Briefly, the relative fractions of the two components, namely I_SOFT_ and I_HARD_, were determined by both estimating the filling factor of the scattering volume for each spectrum in the map and then weighing P_SOFT_ and P_HARD_ intensities for a coefficient measuring their different scattering efficiency, as explained in [26]. Finally, the average elastic modulus M_AVERAGED_ for each spectrum was determined using the Voigt model, considering both the modulus (M_SOFT_ and M_HARD_) and the relative percentage of each peak (I_SOFT_ and I_HARD_) in the scattering volume:

(3) $$M_{\mathrm{AVERAGED}}\;=\;\frac{I_{\mathrm{SOFT}}{\;*\;M}_{\mathrm{SOFT}\;}+{\;I}_{\mathrm{HARD}}{\;*\;M}_{\mathrm{HARD}}}{I_{\mathrm{SOFT}}+{\;I}_{\mathrm{HARD}}}$$

The ratio between the tissue density and the refractive index (ρ/n = 1.29 gr/cm^3^) was considered constant along with the tissue [19,26].

### 2.4. Raman Data Analysis

Raman light scattering arises from the interaction of light with the vibrational modes of the macromolecules present in the sample, thus unveiling its chemical composition. The intensity of Raman peaks depends on the concentration of the chemical species, whilst their frequency shift is due to the chemical nature of the oscillators present in the scattering volume [15]. It is worth noticing that a small shift in the Raman peak position can also be due to the chemical environment in which the oscillator is present, as well as to local variations in the temperature and/or stress acting on it. The thermal loading also influences the line width of the peak by inducing a broadening in the band as the temperature increases, and vice versa [33]. Finally, the heterogeneity of the chemical environment can also affect the line width of the peak by introducing a heterogeneous broadening due to the rise of different contributions very close in frequency. However, in our experimental condition, both thermal and stress effects, which can be very relevant in crystalline or electric conductive materials, are certainly more negligible. Furthermore, as for Brillouin spectroscopy, the overall intensity of the spectrum is also influenced by the quantity of matter enlightened by the laser beam; thus, it is good practice to normalize the Raman intensities to internal standards given by the intensity of different bands [17].

Typical Raman spectra collected in bone tissue are reported in Figure 1b,c (after the break). All the Raman spectra underwent a baseline removal correction via a polynomial fit to reduce the fluorescence background before further calculations. Then, the spectra collected in ROIs were normalized to the CH_2_ wagging signal at 1445 cm^−1^ and used to calculate both the averaged spectrum and the standard deviation from the mean for each frequency detected from about 120 to 4000 cm^−1^, to compare ROI chemical phenotypes.

Raman spectroscopy has been used as a technique for assessing bone quality by the estimation of some important parameters [34,35,36], such as (i) the mineral-to-matrix ratio, evaluating the relative content of hydroxyapatite to the bone organic constituents, which is usually calculated using the intensity of ν_1_PO_4_^3−^ vibration at 965 cm^−1^ and CH_2_ signal at 1445 cm^−1^ or proline at 860 cm^−1^, and (ii) the hydroxyapatite crystallinity, evaluating the matrix ageing through the frequency shift or the line width at half height of ν_1_PO_4_^3−^ vibration. In our case, we chose the relative ratio between ν_1_PO_4_^3−^ and proline intensities as a marker for collagen bundle mineralization degree. In addition to this, the first spectral moment of the multiple-peaked CH_2_-CH_3_ band at 2800–3100 cm^−1^ was used to evaluate the lipids-to-proteins ratio. A shift to lower frequency is expected in this band, when a higher contribution from the CH_2_ stretching of lipids at 2872 cm^−1^ to the CH_3_ stretching of proteins at 2935 cm^−1^ occurs, as reported in Figure 1b,c [17]. Similarly, the first spectral moment of the band between 1500 and 1720 cm^−1^ was calculated to estimate the contribution of heme groups at 1580 cm^−1^ [37] to proteins and unsaturated fatty acid signals at 1660 cm^−1^ and to mark the region with a prevalence of red bone marrow on the extracellular matrix component and the adipocytes [26,38]. In both cases, the first spectral moment was calculated using Equation (2), with i spanning from 2800 to 3100 cm^−1^ and from 1500 and 1720 cm^−1^, respectively. Table 1 summarizes some of the typical Raman vibrations detectable on both cortical and trabecular bone tissues.

#### PCA Analysis of Raman Spectra

To deepen the chemical analysis of bone tissue, Raman spectra from the ROIs were processed using also a multivariate statistical approach, namely principal component analysis (PCA) [27,28]. PCA is an unsupervised method used to reduce the dimensionality of the dataset by identifying principal components—i.e., combinations of variables that are more involved in the description of the dataset variance. The contribution of each variable (i.e., the Raman frequency) to the principal component is defined as loading, while the position of each observation (i.e., the Raman spectrum) in this new coordinate system of principal components is defined as a score. Different data pretreatments and intervals of frequencies were tested to obtain the best PCA classification, revealing that the spectra baseline removed and normalized to the CH_2_ wagging signal at 1445 cm^−1^ is the most effective, especially when considered in the so-called fingerprint region between 800 and 1780 cm^−1^. PCA analysis was performed in the RStudio environment (www.rstudio.com accessed on 30 September 2021), using the R built-in function prcomp in the base package, whereas the score plots were obtained using the Plotly graphical package. It is worth noting that the multivariate statistics was applied only to Raman spectra, due to their extraordinary richness in terms of spectral features retrieved from different regions of the samples.

## 3. Results

### 3.1. Chemo-Mechanical Imaging of a Human Femoral Head Section

Figure 1b,c report typical Brillouin and Raman spectra collected on ROIs of the trabecular bone (red square) and cortical bone (black square), excised from the femoral epiphysis, as described above in Section 2.1. In both cases, Brillouin peaks (before the break) show the coexistence of at least two Brillouin regions in the same spectrum, P_SOFT_ that ranges between 4 and 13 GHz and P_HARD_ that includes between 13 and 32 GHz. In contrast, the typical Raman signatures (after the break) reveal the presence of diverse contributions in the tissue types, especially in the content of organic constituents—i.e., proteins and lipids—as well as an evident variation in the hydroxyapatite content. In our previous work [26], we inferred that this mechanical heterogeneity at the microscale is due to the presence of two distinct ECM organizations in the lamellar bone—i.e., the not-ordered and ordered phase—also predicted by Reznikov et al. in [2,5]. Specifically, the P_SOFT_ component originates from both the loose phase of ECM, composed of water, non-collagenous proteins, and poorly oriented fibres in the cortical bone tissue, and the bone marrow substance encapsulated among the bony spiculae in the trabecular bone [39], whereas the P_HARD_ component originates from the aggregates of mineralized collagen bundles. Brillouin and Raman mapping, performed on the same ROIs, allows appreciating the spatial distribution of the main chemo-mechanical signatures found, adding important pieces of information to the solely single-spectrum analysis.

**Figure 2:**
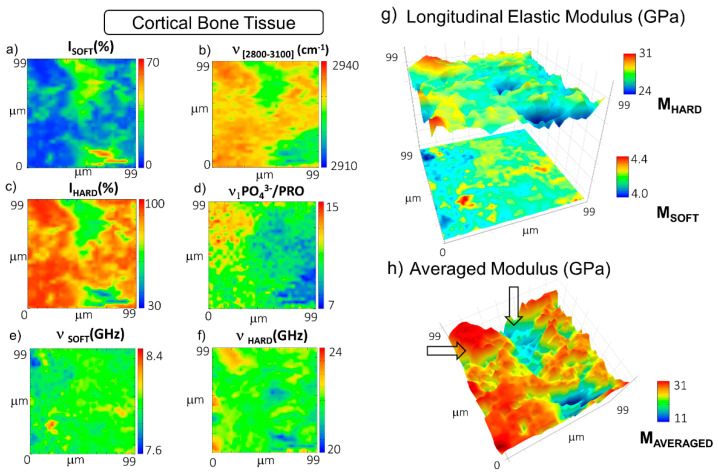
Maps of the distribution in the cortical bone of (**a**) the percentage of P_SOFT_ component; (**b**) the first spectral moment of CH_2_-CH_3_ signal at 2800–3100 cm^−^^1^; (**c**) the percentage of the P_HARD_ component; (**d**) the mineralization degree expressed as the relative ratio between the ν_1_PO_4_^3−^ signal at 965 cm^−^^1^ and the proline signal at 860 cm^−^^1^; the frequency shifts of (**e**) the P_SOFT_ and (**f**) the P_HARD_ components; (**g**) stacked surface plots of the longitudinal elastic moduli of the soft and hard components (M_SOFT_ and M_HARD_); (**h**) surface plot of the average longitudinal elastic modulus (M_AVERAGED_). Arrows point out areas mainly occupied by the ECM (higher values in red) and by the central blood vessel (lower values in blue).

Figure 2a–f report the maps of the spatial distribution of Brillouin–Raman intensities and frequency shifts of peaks of interest in the bone tissue spectra, calculated using the spectral moments as described in Materials and Methods. The Brillouin relative percentages of the volume occupied by the P_SOFT_ and P_HARD_ components, namely I_SOFT_ and I_HARD_, reported, respectively, in Figure 2a,c were calculated using the procedure previously described in Materials and Methods and [26]. Moreover, the relative ratio between PO_4_^3−^ and proline Raman intensities was chosen as a marker of the mineralization content of the collagen fibres present in the tissue, whereas the first spectral moment of the CH_2_-CH_3_ band at about 2800–3100 cm^−1^ was determined to express the lipid-to-protein ratio. Finally, Figure 2g reports the longitudinal elastic moduli, M_SOFT_ and M_HARD_, calculated for the P_SOFT_ and the P_HARD_ components, whereas in Figure 2h, the tissue averaged modulus M_AVERAGED_ was obtained using the procedure explained in Materials and Methods (Section 2.3).

The distribution of the not-ordered phase of bone in the cortical bone, i.e., the I_SOFT_ map in Figure 2a, most likely reveals the presence of two central blood vessels, whereas the distribution of the ordered phase of collagen bundles, i.e., the I_HARD_ map in Figure 2c, depicts the extracellular mineralized matrix forming the lamellar portion of the bone [39]. The relative percentages of the two components highlight that this ordered phase is the main ECM organization present in the cortical bone. According to this interpretation of the Brillouin peaks, the Raman frequency shift of the CH_2_-CH_3_ band shows lower values in the central channel, likely due to the occurrence of lipidic residues in cellular membranes, and higher values in the extracellular matrix due to the massive presence of collagen fibres. Figure 2e maps the distribution of the P_SOFT_ frequency shift, ν_SOFT_ (i.e., the not-ordered phase stiffness), revealing an almost homogenous modulus. Finally, Figure 2f unveils the distribution of the P_HARD_ frequency shift, ν_HARD_ (i.e., the hard-phase stiffness), unveiling a certain correspondence with the Raman signature for the matrix mineralization degree in Figure 2d and disclosing a behaviour similar to that of the cortical region of the femoral diaphysis previously reported in [26].

Figure 2g reports the surface plots of the longitudinal elastic modulus of both soft and hard components, M_SOFT_ and M_HARD_, obtained through Equation (1), unveiling that the not-ordered phase of the extracellular matrix has a modulus ranging from about 4.0 to 4.4 GPa, while the mineralized extracellular matrix retains higher values, going from about 24 to 31 GPa. It should be stressed that the possibility to discriminate the contributions of these two components is a peculiarity of Brillouin spectroscopy since other techniques suitable for mechanical characterization at the microscale provide only averaged values. In addition to the separated components, the spatial distribution of the averaged longitudinal elastic modulus, M_AVERAGED_, was calculated using Equation (3) for every single point of the map (Figure 2h), which takes into consideration not only the frequency shift of the single elastic species but also their relative concentrations in the scattering volume, thus providing information about the tissue’s overall elastic properties. M_AVERAGED_ values span a wide range from about 11 to 31 GPa, revealing through their spatial distribution that even if the average modulus of the cortical bone is dominated by the contribution of the stiff mineralized phase, the central blood vessels can still be easily recognized on the map, due to their considerably lower elastic modulus (arrows in Figure 2h).

**Figure 3:**
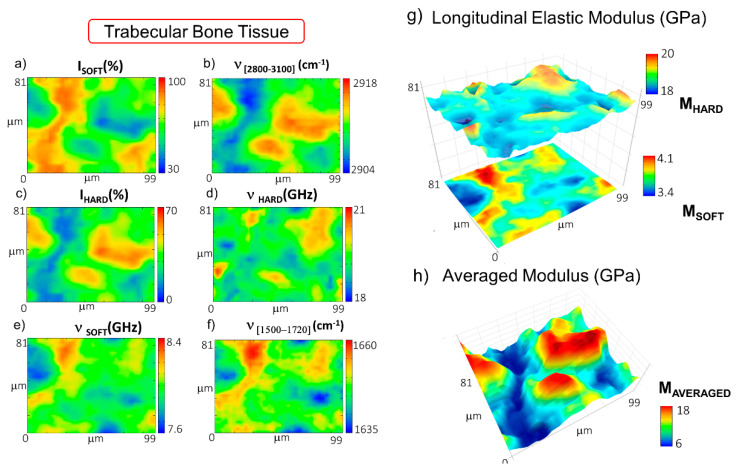
Maps of the distribution in the trabecular bone of (**a**) the relative percentage of P_SOFT_ component; (**b**) the first spectral moment of CH_2_-CH_3_ signal at 2800–3100 cm^−^^1^; (**c**) the relative percentage of the P_HARD_ component; the frequency shifts of (**d**) the P_HARD_ and (**e**) the P_SOFT_ components; (**f**) the first spectral moment of the heme-amide I signal at 1500–1720 cm^−^^1^; (**g**) stacked surface plots of the longitudinal elastic moduli of the soft and hard components (M_SOFT_ and M_HARD_); (**h**) surface plot of the average longitudinal elastic modulus (M_AVERAGED_).

A similar procedure was applied to the mapping of the trabecular bone region of the femoral epiphysis. In particular, Figure 3a–f reports the spatial distribution of the relative percentage of the volume occupied by the soft and the hard components, I_SOFT_ and I_HARD_, with their frequency shifts, ν_SOFT_ and ν_HARD_. In addition to this, the frequency shift of the CH_2_-CH_3_ band is reported to map the lipid-to-protein ratio, whereas the frequency shift of the band at about 1500–1720 cm^−1^ is employed to identify the presence of blood inside the tissue (see Materials and Methods). Figure 3g reports the longitudinal elastic moduli, M_SOFT_ and M_HARD_, calculated for the P_SOFT_ and the P_HARD_ components, respectively, and Figure 3h reports the tissue averaged modulus M_AVERAGED_. In this case, the distribution of the I_SOFT_ component (Figure 3a) unveils areas of tissue encapsulated between wide regions mainly occupied by the I_HARD_ constituent—i.e., the mineralized matrix (Figure 3c). This interpretation of the Brillouin data is also confirmed by the trend of the Raman band at 2800–3100 cm^−^^1^ in Figure 3b, which shows a shift to a higher content of lipids in the region occupied by I_SOFT_ (i.e., the non-order phase) and a shift to higher protein content in the areas characterized by higher values of I_HARD_ (i.e., the order phase). This mesh of soft and hard portions is probably due to spicules of lamellar bone intermixed with regions containing the bone marrow constituents. Moreover, the range of values assumed by ν_HARD_ (Figure 3d) and the Raman mineralization degree (data not shown) was found to be lower than those evaluated in the cortical tissue. These pieces of evidence agree with the concept that trabecular bone tissue is designed (i) to provide support to the marrow, (ii) to regulate calcium and phosphate ion homeostasis, and (iii) to transfer the load from joints to the compact bone of the cortex of long bones [40]. Thus, the result can potentially come out in a more disordered and less mineralized collagen bundle architecture, likely due to bone remodelling processes [4].

Figure 3e reports the distribution of ν_SOFT_ values, revealing that some portions of the soft component of the tissue are characterized by even lower values of the modulus. The origin of this gradient can be related to the presence of red bone marrow constituents, as disclosed by the first spectral moment of the Raman band at 1500–1720 cm^−^^1^ shown in Figure 3f. In fact, a shift to lower values in the map identifies a higher concentration of hemoglobin content with respect to proteins and fatty acids (see Materials and Methods). The longitudinal elastic modulus of the soft and hard components, M_SOFT_ and M_HARD_, is reported in Figure 3g, showing values going from 3.47 GPa to 4.10 GPa and from 18.25 GPa to 20.86 GPa, respectively. The average longitudinal elastic modulus M_AVERAGED_ in Figure 3h unveils variations ranging between 6 GPa in the regions occupied by the red bone marrow constituents and 18 GPa in the regions filled by the bony spicules. It is worth noting that the average elastic modulus is lower than the one found in the cortical lamellar bone, confirming previous findings in the literature [41,42,43,44].

### 3.2. Effects of Sample Processing on the Structural and Mechanical Properties of Bone Tissue

**Figure 4:**
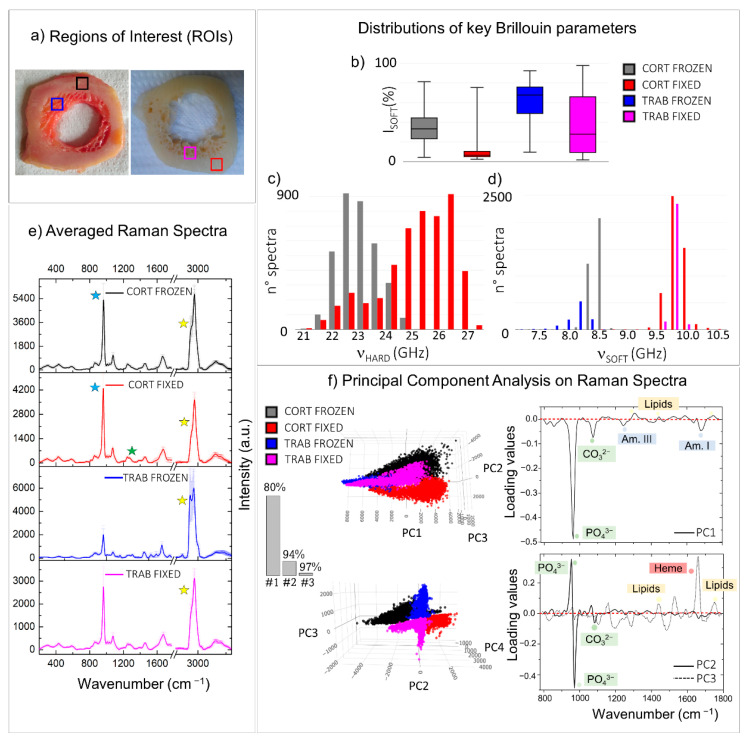
Comparison between regions of interest (ROIs) collected from two similar histological sections treated with different preparation processes. (**a**) Pictures of two diaphyseal cross-sections treated with two distinct sample preparation processes: freezing at −80 °C (on the left) and PFA-fixation followed by ethanol storage (on the right). The coloured squares enclose ROIs selected for comparisons (black: frozen cortical bone; red: fixed cortical bone; blue: frozen trabecular bone; magenta: fixed trabecular bone). Box plots and histograms report the statistical distributions of relevant Brillouin parameters obtained from the ROIs, namely (**b**) the relative percentage of the P_SOFT_ component, (**c**) the frequency shift ν_HARD_ of the P_HARD_ component, and (**d**) its frequency shift ν_SOFT_. (**e**) Average of all the normalized Raman spectra obtained from the ROIs. The y-error bars represent the standard deviation of Raman intensities calculated for each of the four datasets. (**f**) Score plots and loading plots of the principal component analysis performed on the Raman spectra of ROIs. Coloured labels in the loading plots are used to stress the presence of spectral features referable to a certain class of macromolecules. The histogram reports the cumulative variance expressed by the first (1#), the second (#2), and the third (#3) principal components.

All samples investigated up to now by micro-Brillouin and Raman techniques have been subject to preservation treatments. It is thus important to investigate the effect of sample processing on bone chemo-mechanical properties [45]. In Figure 4, we use the knowledge previously acquired to compare the chemo-mechanical properties of two cross-sections of femoral diaphysis, subjected to different sample treatments. Specifically, the section on the left panel of Figure 4a was frozen at −80 °C, whereas the section on the right was fixed in PFA and then stored in ethanol (for further details, see Materials and Methods). The coloured squares enclose regions of interest (ROIs) in the cortical (black and red squares) and in the trabecular bone (blue and magenta squares).

Distributions of relevant Brillouin parameters evaluating the averaged mechanical properties of tissue on frozen and fixed conditions were reported in Figure 4b–d. Raman analysis was performed using both a traditional approach, namely the comparison between the averaged spectra of the four regions of interest, and an unsupervised statistical method—i.e., principal component analysis (PCA), usually used to reduce the dimensionality of a dataset by the extraction of principal components (PCs). Specifically, the averaged spectra obtained from the mean of thousands of measurements collected in each of the four ROIs were reported in Figure 4e, together with the standard deviations of the intensities for each frequency from 280 to 3660 cm^−1^ (y-error bars), revealing the intra-chemical variability within each of the regions analysed. On the other hand, the PCA scatter plots (i.e., the coordinates of each spectrum in the reduced space) and the PCA loading plots (i.e., the weighted contributions of the variables to each PC) of the first three principal components are shown in Figure 4f, along with a histogram showing the explained cumulative variance. In both cases, Raman spectra were baseline removed and normalized to the CH_2_ wagging signal at 1445 cm^−1^, prior to other computations (further details are provided in Materials and Methods).

The box plot of Figure 4b represents the relative percentages of the volume occupied by the P_SOFT_ constituents in the four ROIs, disclosing a higher presence of I_SOFT_ in the Brillouin spectra of frozen tissues (black and blue boxes) than the fixed ones (red and magenta boxes) for both the cortical and the trabecular bone. In particular, the frozen trabecular bone retains the highest soft contribution among the four different regions analysed. At the same time, the Raman spectral analysis conducted through the comparison of the ROI-averaged spectra (Figure 4e) shows that the averaged cortical bone spectra of the frozen (black spectrum in Figure 4e) and the fixed (red spectrum), almost similar, present small variations in the CH_2_-CH_3_ stretching region between 2800 and 3100 cm^−1^ (yellow stars), revealing a higher lipid-to-protein ratio in frozen tissue. A similar behaviour but with greater differences can be appreciated in the averaged trabecular bone spectra of the frozen (blue spectrum) and the fixed (magenta spectrum) ROIs. Specifically, in the trabecular region of the frozen sample, Raman spectra are characterized by elevated intra-variability, well-visible in the standard deviations of the CH_2_-CH_3_ band, and a higher content of lipids than the fixed tissue (yellow stars). Likewise, the PCA scores plot in the top of Figure 4, panel f, reveals that the first principal component (PC1) divides the spectra collected in the frozen trabecular bone (blue) from the great majority of the spectra collected in the other three ROIs. The respective loading plot on the right provides information on the chemical origin of its diversity from the other groups: the spectra collected in the frozen trabecular bone (blue points) have a higher concentration in lipid content with respect to proteins, as well as a lower contribution from mineralized bundles.

Figure 4c reports the histogram of the ν_HARD_ distribution in the frozen cortical bone (black) and the fixed cortical bone (red), revealing higher values in the stiffness of the ordered phase of mineralized collagen bundles in the fixed sample, corresponding to a difference of about 15% in the elastic constants. Based on the previous evidence, this fact can likely be due to a higher fibre mineralization degree. However, it is reasonable that increased stiffness in the fibres could also be partially attributed to the fixation procedure using PFA since it introduces cross-links into the collagen fibrillar structure [45,46]. The Raman investigation performed in the same regions seems to suggest that the increased stiffness can be due to a combination of both these factors. In fact, if, on the one hand, the averaged cortical bone spectrum of the fixed ROI (red spectrum in Figure 4e) shows a mineral-to-matrix ratio (blue stars) higher than that of the frozen ROI (black spectrum), on the other hand, small variations in the amide III spectral shape can be observed (green star). This result also agrees with the PCA scatter plot of the second (PC2) principal component, reported at the bottom of Figure 4, panel f, with its respective loading plot. Interestingly, PC2 divides the spectra collected in the frozen cortical bone (black points) from the ones in the fixed cortical bone (red points), revealing that the v_1_PO_4_^3−^ vibration of the phosphate group shows a slight shift to higher values in the fixed sample, together with mild differences in the shape of both the amide I and amide III bands.

Finally, in Figure 4d, the histogram of the ν_SOFT_ distribution is reported for the four ROIs, revealing a shift to higher values of ν_SOFT_ in fixed tissues (red and magenta), to the frozen ones (black and blue), corresponding to an increase of about 34% and 37% in the elastic constants in cortical and trabecular tissue types, respectively. This fact can be a consequence of the different chemical nature of the P_SOFT_ constituents in the frozen and the fixed sample: the preservation of the cellular component and bone marrow constituents causes a decrease in the modulus of the not-ordered phase P_SOFT_ as reported in Section 3.1 and [26]. On the other hand, in fixed tissues, the removal of the lipid component leaves exposed a mesh of non-collagenous proteins, disorganized and undirected collagen fibres, and water, characterized by slightly higher values of frequency shift [47]. These results are confirmed by Raman spectral analysis, using both the averaged spectra shown in Figure 4e, where the frozen tissues reveal a higher signal from the CH_2_ stretching of lipids at 1845 cm^−1^ (yellow stars), heme and carotenoid groups (red stars), and the scatter plot of the third principal components (PC3) in Figure 4f, bottom panel. In fact, PC3 generally divides the spectra collected in the frozen tissues (black and blue) from the fixed ones (red and magenta), disclosing that the frozen tissues are characterized by the presence of lipids (cell membranes and vessels), carotenoids, and heme groups (red and yellow bone marrow constituents), while the proteins are the main constituents of the loose ECM in the fixed sample, as reported in the loading plot on the right of Figure 4f.

### 3.3. Differences in the Structural and Mechanical Properties of Cortical and Trabecular Bone Excised from Different Anatomical Regions

**Figure 5:**
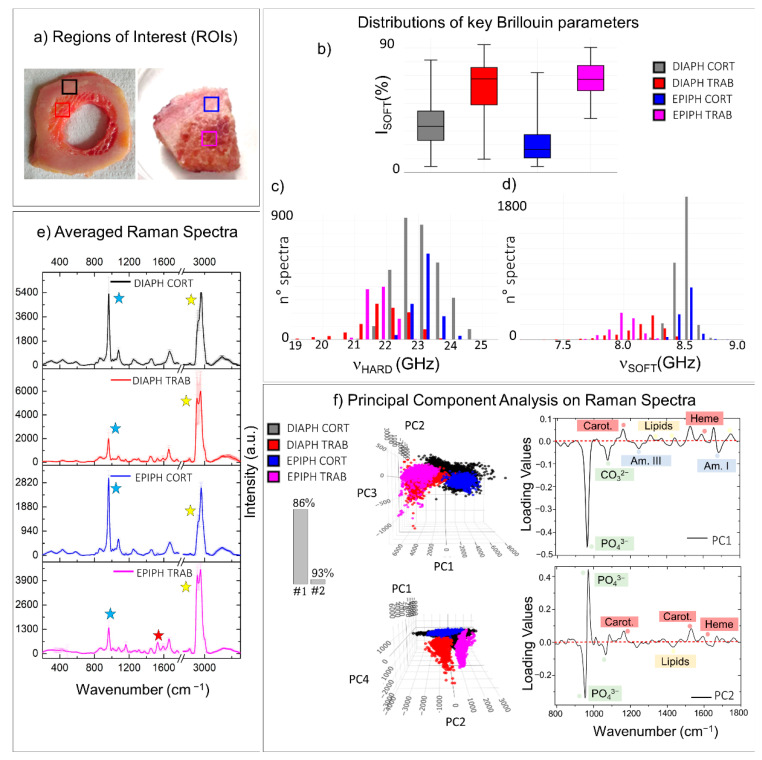
Comparison between regions of interest (ROIs) collected from cortical and trabecular bone tissue types belonging to different anatomical portions. (**a**) Pictures of two different tissue sections both excised from the human femur and then frozen at −80 °C: a cross-section of the diaphysis and a section from the infero-medial epiphysis (on the right). The coloured squares enclose ROIs selected for comparisons (black: diaphyseal cortical bone; red: diaphyseal trabecular bone; blue: epiphyseal cortical bone; magenta: epiphyseal trabecular bone). Box plots and histograms report the statistical distributions of relevant Brillouin parameters obtained from the ROIs, namely (**b**) the relative percentage of the P_SOFT_ component, (**c**) the frequency shift ν_HARD_ of the P_HARD_ component, and (**d**) its frequency shift ν_SOFT_. (**e**) Average of all the normalized Raman spectra obtained from the ROIs. The y-error bars represent the standard deviation of Raman intensities calculated for each of the four datasets. (**f**) Score plots and loading plots of the PCA performed on the Raman spectra of ROIs. Coloured labels in the loading plots are used to stress the presence of spectral features referable to a certain class of macromolecules. The histogram reports the cumulative variance expressed by the first (1#) and the second (#2) principal components.

Figure 5 illustrates the results of the comparison of the chemo-mechanical characteristics of cortical and trabecular tissue types, belonging to different anatomical portions of the human femur, i.e., the diaphysis and the epiphysis, aiming to verify the origin of mechanical variability among the different portions of the same type of bone [48,49]. The same sample treatment was chosen for both sections to avoid alterations due to external factors. Specifically, the freezing procedure at −80°C was selected since it has proven to maintain properties closer to the native tissue. The cortical bone, which forms the strong outer scaffold of the long bones, constitutes both the diaphyseal ring (Figure 5a: left side-black box) and the first portion of the subchondral bone of the epiphysis (Figure 5a: right side-blue box). Similarly, the trabecular bone extends into the medullary cavity of the diaphysis (Figure 5a: left side-red box) and the innermost portion of the subchondral bone of the epiphysis (Figure 5a: right side-magenta box). The portions in the coloured squares were selected as regions of interest (ROIs) to perform the comparison between the different tissue organizations. Figure 5b–d reports distributions of relevant Brillouin parameters, while Raman analysis was performed using both the traditional approach, namely the comparison between the averaged spectrum collected in the four regions of interest (Figure 5e) and the PCA method (Figure 5f), as explained in the previous section. In the PCA analysis, the first two principal components (#1 and #2) are effective in classifying different phenotypes, accounting for 93% of the spectral variance in the dataset.

The box plot of Figure 5b reports the I_SOFT_ component distribution in the four ROIs, showing that it is noticeably higher in the trabecular bone tissue than the cortical bone, while the ν_HARD_ distribution reported in the histogram of Figure 5c clearly shows lower values of frequency in the mineralized collagen bundles of trabecular bone than the cortical one, thus confirming the previous findings shown in the tissue mapping in Section 3.1. This result is also evident in the Raman-averaged spectra of the ROIs in Figure 5e, where the spectra obtained in the cortical bone of both the diaphysis and the epiphysis are characterized by lower content in lipids (yellow stars) and a higher mineralization degree (blue stars), with respect to the trabecular tissues. Likewise, the PCA scatter plot at the top of Figure 5f shows that the first principal component (PC1) divides the spectra characteristics of the cortical bone from the one typical of the trabecular bone for both diaphysis (black and red) and epiphysis (blue and magenta). The respective loading plot on the right confirms that the cortical spectra marked by the black and blue points show a prevalence of peaks coming from the ordered mineralized matrix. Otherwise, a higher contribution from the heme group and lipid peaks is characteristic of red and magenta points, namely markers of trabecular bone spectra. It is worth noting that PC1 also retains some information about the main difference between the chemical composition of the diaphyseal cortical bone (black points) to the epiphyseal cortical region (blue points). Specifically, the black scores in the PCA space depict a wide cluster, thus revealing higher chemical heterogeneity than the spectra collected in the cortical bone of the subchondral bone, which is homogeneous and characterized by a higher average content of hydroxyapatite. This chemical heterogeneity is also reflected in the higher mechanical anisotropy of the diaphyseal region (black) compared to the epiphysis region (blue), as shown in the histogram of Figure 5c reporting the distribution of the ν_HARD_ component. These differences suggest that the cortical bone tissue can be differently organized in the two anatomical regions, likely due to different mechanical functions [49].

Moreover, the histogram of Figure 5d, showing the distribution of ν_SOFT_ values, reveals (i) a lower-frequency shift in trabecular tissues compared with the cortical ones and, among the trabecular tissues, (ii) a lower-frequency shift in the epiphyseal region compared with the medullary canal. The chemical origin of these differences is disclosed by both the averaged spectra of ROIs in Figure 5e and the PCA analysis in Figure 5f. In fact, the average spectrum of trabecular tissues contains a higher content in lipids with respect to the cortical ones (yellow star), and more importantly, the Raman spectrum of the trabecular bone in the epiphyseal regions retains higher contributions from the blood-series constituents, such as heme groups and carotenoids, when compared to the trabecular bone in the medullary cavity (red stars). Likewise, the second principal component (PC2) divides the spectra collected in the trabecular bone of epiphysis (magenta) from the one collected in the medullary cavity of the diaphysis (red). Its loading plot in the bottom right part of Figure 5f confirms a higher contribution from the heme and carotenoids groups in the epiphyseal region than the diaphyseal one, instead of showing higher content in lipids. A possible explanation of this result can be found in the fact that bone marrow encapsulated among the trabeculae can be constituted by both a yellow and a red part, depending on the main contributors from either lipids or blood precursors, respectively [4].

## 4. Discussion

Bone tissue shows a complex structure with a sophisticated hierarchical architecture that already starts at the microscale [5]. In this work, we showed how the BRmS technique can give access to structural and mechanical information at the micro-scale in a non-invasive and non-destructive way, both on cortical and trabecular lamellar bone. However, this study presents some limitations, mainly related to a small number of analysed samples and their donor-dependent variability. Nevertheless, the specimens analysed with the BRmS technique were obtained from musculoskeletal tissues procured from cadaver donors. Each donor was selected by strict criteria as issued by the National Transplant Centre’s guidelines [50] to guarantee the safety and efficacy of procured, processed, and distributed tissues for transplant purposes. Therefore, in these selected donors, the absence of metabolic, neoplastic, infective, and degenerative bone diseases is noticeable, which contributes to mitigating donor-related inter-variability. However, categorical variables, such as age or sex, must be also taken into account. Hence, although the proof of concept presented here has shown promising results, further analyses on a larger number of samples are needed to achieve a more detailed and comprehensive characterization of bone architecture by BRmS protocols. The different forms of sub-organization of the extracellular matrix have been analysed, i.e., the ordered and the not-ordered phases, revealing the possibility to monitor not only the shape and distribution of the main constituents of the lamellar bones but also their chemical composition and longitudinal elastic modulus. More specifically, the analysis of the averaged elastic modulus M_AVERAGED_ has revealed a range of values from 11 to 31 GPa and a value from 6 to 18 GPa for the frozen cortical and trabecular bone in the epiphyseal region, respectively, confirming the wide heterogeneity of bone tissue structural properties and mechanical performance, also found by other groups [40,41,42,43,44]. It is worth noting that the majority of the results reported in the literature referred to the application of nanoindentation and the acoustic technique, extracting the tissue’s Young’s moduli (E) instead of the longitudinal elastic modulus (M). Nevertheless, since in the solid-like sample, the viscoelastic mechanisms are less relevant, the elastic moduli measured by Brillouin spectroscopy are expected to be in the same order of magnitude. Moreover, it should be stressed that Brillouin spectroscopy allows monitoring not only the average values of the modulus but also the local modulus for each biological constituent in bone, providing a unique tool for mechanics investigation at the microscale. In detail, the longitudinal elastic moduli of the soft portion, i.e., the cellular phase of osteocytes (M_SOFT_), and the hard portion, i.e., the mineralized bundles (M_HARD_), show values going from 4.0 GPa to 4.4 GPa, and from 24 GPa to 31 GPa, respectively, in the cortical bone, whereas in the trabecular bone, the cellular constituents (adipocytes and erythrocytes) reach even smaller values of about 3.47 GPa to 4.10 GPa, as well as the mineralized extracellular matrix which shows values going from about 18.5 GPa to 20.86 GPa. It is worth noting that in our previous work [26], which focused on mapping a different anatomical portion of the femur (i.e., a cross-section of the diaphysis), although similar Brillouin and Raman spectral types were found going from the outermost and innermost portions of the bone, both structural and mechanical differences were discovered, likely due to the dissimilar landscape of chemical and mechanical forces acting on these two anatomical portions of the bone.

Moreover, the employment of multivariate statistical approaches, such as PCA, to the spectral analysis has proven to be effective in highlighting differences in the chemical composition and their influence on the mechanical properties of bone tissue when subjected to different treatments, such as fixation in PFA followed by storage in ethanol or freezing at −80 °C. More specifically, bone tissues treated with fixation in PFA and ethanol were found to (i) undergo a massive loss of the lipid component, especially in the fatty acid content in the trabecular tissue with respect to the cortical one, and (ii) display small variations in the bands referring to proteins and hydroxyapatite vibrations. These findings agree with the fact that the freezing procedure preserves the fatty acids and esters in the tissue from the solvation processes due to the treatment with alcohols, thus protecting the cellular constituents of bone (osteocytes, adipocytes, and red cells) [47], and at the same time, they suggest the presence of small variations in the structure of the mineralized collagen fibres since the amide peaks are sensitive to the secondary and tertiary structures of collagen, as well as the v_1_PO_4_^3−^ frequency shift to the crystalline structure of hydroxyapatite crystals. A possible explanation is that although the fixation process with PFA introduces cross-links between the residues of some amino acids in the collagen fibres [45,46], a mild distortion in the mineralized bundles can be introduced, producing variations in the local chemical environment of the hydroxyapatite crystals. These results underline how the freezing procedure is less invasive than the fixation in PFA followed by preservation in ethanol, as it allows the preservation of both the lipidic constituent and the collagen structure. All these factors contribute to an increase in the tissue average elastic modulus (M_AVERAGED_) from 22.40 ± 0.08 GPa to 34.20 ± 0.08 GPa (cortical tissue) and from 13.40 ± 0.16 GPa to 24.00 ± 0.10 GPa (trabecular tissue), obtained by computing the mean of the averaged moduli obtained from each spectrum in the ROIs of Figure 4. However, this difference, which corresponds approximately to a substantial increase of about 53% and 79% in the average modulus (M_AVERAGED_) of cortical and trabecular tissue, respectively, in the case of fixed versus frozen specimens, is mainly ascribable to the depletion of the lipids, caused by the storage in ethanol [46], rather than to a stiffening of the mineralized collagen bundles due to fixation in PFA. In fact, the ethanol storage causes a loss of about 70% and 60% in the concentration of the P_SOFT_ elastic species in the cortical and trabecular tissue types, with a simultaneous increase of about 34% and 37% in its averaged modulus M_SOFT_. Conversely, the effect of PFA fixation on the M_HARD_ value is considerably less: the increase in the elastic modulus, in fact, even assuming that it is due exclusively to the fixing procedure and not to endogenous differences in the fibres’ degree of mineralization, does not exceed the 15% and 4%, respectively, for cortical and trabecular tissue. Consequently, Brillouin scattering (BLS), thanks to the advantage of singling out the elastic constants of both hard (mineralized) and soft sub-micrometric portions of the bone, can provide mechanical information less affected by sample treatment with respect to less spatially resolved techniques. This ability to distinguish and characterize the mechanical properties of the hard micro-regions, with minor dependence on preservation protocols, paves the way to the use of this technique for the study of some critical issues in bone research, starting from comfortably fixed samples.

Having established that the freezing procedure is less invasive for tissue analysis, the same statistical analysis has been used to compare the chemo-mechanics of bone regions with a similar macroscopic organization (cortical and trabecular bone) but different anatomical localizations (epiphysis and diaphysis). Several works in the literature have shown differences in the elastic moduli of sections excised from different anatomical regions of the same bone, such as the femoral neck and the midshaft diaphysis, or the proximal and distal bones [48,49]. It is worth noting that in our case, the femoral head section was excised from the infero-medial region, a portion of bone proximal but not coincident with the femoral neck. The results reveal that the cortical bone forming the cross-section of the diaphysis retains a certain mechanical anisotropy due to the lamellar pattern, resulting in a higher heterogeneity with respect to the cortical bone tissue excised from the subchondral bone plate, which is more homogenous and more highly mineralized. This result is in accordance with the notion that the cortical lamellar bone placed in the subchondral region has to cope with high mechanical stress due to the proximity of the articulation with the hip compared to the diaphyseal part of the femur.

However, it is the trabecular tissue organization obtained from the femoral epiphysis and the medullary canal of the diaphysis that shows the greatest differences since the areas among the epiphyseal trabeculae are marked by signals from the red and yellow bone marrow, while in the medullary canal, mainly signatures from the adipocytes can be retrieved. This result agrees with the fact that during growth, the red bone marrow in the medullary cavity is almost totally replaced by adipocytes, thus transforming into yellow adipose tissue, while the hematopoietic cells are preserved only in the epiphyseal region of long bones [4]. This different chemical composition results in a variation of the average elastic modulus (M_AVERAGED_) of the cortical tissue from 22.4 ± 0.08 GPa (diaphysis) to 26.3 ± 0.11 GPa (epiphysis) and from 13.4 ± 0.16 GPa (medullary cavity) to 11.6 ± 0.09 GPa (epiphysis) for the trabecular bone, obtained by the calculation of the mean of M_AVERAGED_ values of each spectrum in the ROIs of Figure 5. These variations correspond to an increase of approximately 17% in the averaged stiffness of the cortical tissue excised from the femoral epiphysis with respect to the femoral diaphysis and a simultaneous decrease of about 13% in the stiffness of trabecular tissue excised from the femoral epiphysis to the one collected from the medullary cavity in the diaphysis. A summary of the main results found in the comparison of mean values of the longitudinal elastic moduli, between the cortical and trabecular bone excised from the different portions of the femur (diaphysis and epiphysis) and treated with different sample preparations (freezing and fixation/ethanol storage), is provided in Table 2.

## 5. Conclusions

The BRmS analysis of healthy sections of bones merged with powerful methods based on multivariate statistical approaches presented here constitutes the basis for any further application of this technique to the characterization of bone tissue micro-architecture and mechanics alterations from the native phenotype.

We have shown that the BRmS analysis can give important insights into the bone microstructure in both the cortical and the trabecular tissue organizations, revealing the chemo-mechanical phenotype of Haversian channels and bone marrow constituents (soft component), as well as of the mineralized extracellular matrix forming the lamellar substance (hard component). Moreover, PCA analysis applied to the chemical phenotype, aided by the evaluation of the longitudinal elastic moduli of both the soft and the hard phases of bone, was applied to discriminate cortical and trabecular bone tissues collected from different anatomical regions and/or subjected to different sample treatments, showing that the simultaneous investigation of both the tissue chemistry and the mechanics guarantees the unique possibility to unveil the chemical origin of potential mechanical deviations in single biological constituents from their native or healthy condition.

Finally, although further studies are needed to confirm the biomedical applications of BRmS, the promising results presented here justify efforts to promote its future development and implementation as a feasible, tuneable, and cost-effective tool for the diagnosis of pathological alterations of musculoskeletal tissues, such as bone cancers or age-related diseases, e.g., osteoporosis and osteoarthritis.

## Figures and Tables

**Table 1:** Typical Raman vibrations detected on bone tissue spectra.

Wavenumber (cm^−1^)	Assignments
860	Proline (benzene ring breathing)
870	Hydroxyproline (benzene ring breathing)
965	ν_1_ PO_4_^3−^ (P-O symmetric stretch)
1003	Phenylalanine (aromatic ring breathing)
1030	ν_3_ PO_4_^3−^ (P-O asymmetric stretch)
1068	ν_1_ CO_3_^2−^ (C-O in-plane stretch)
1125–1160	C-CH_3_ carotenoids
1170	Hemoglobin
1242	Amide III (C-N-H stretch)
1298	Fatty Acids (CH_2_-CH_3_ twisting and wagging)
1445	CH_2_ (lipids)
1580	Hemoglobin
1655–1675	Amide I (C=O stretch)
1745	Esters (C=O)
2872	CH_2_ lipids
2935	CH_3_ proteins
3150–3500	OH stretching

**Table 2:** Summary of the mean values of longitudinal elastic moduli detected on the different sections.

Type of Tissue	Treatment/Anatomical Region	M_SOFT_ (GPa)	M_HARD_ (GPa)	M_AVERAGED_ (GPa)
Cortical bone	Frozen epiphysis	4.310 ± 0.002	31.90 ± 0.03	26.30 ± 0.11
	Frozen diaphysis	4.290 ± 0.002	31.70 ± 0.03	22.40 ± 0.08
	Fixed diaphysis	5.750 ± 0.002	37.40 ± 0.05	34.20 ± 0.08
Trabecular bone	Frozen epiphysis	3.770 ± 0.003	27.70 ± 0.03	11.60 ± 0.09
	Frozen diaphysis	3.950 ± 0.006	28.70 ± 0.06	13.40 ± 0.16
	Fixed diaphysis	5.430 ± 0.005	29.90 ± 0.08	24.00 ± 0.10

## Data Availability

The data presented in this study are available on request from the corresponding author.
